# Oral Anticoagulation in Atrial Fibrillation: Development and Evaluation of a Mobile Health Application to Support Shared Decision-Making

**DOI:** 10.5935/abc.20170181

**Published:** 2018-01

**Authors:** Laura Siga Stephan, Eduardo Dytz Almeida, Raphael Boesche Guimarães, Antonio Gaudie Ley, Rodrigo Gonçalves Mathias, Maria Valéria Assis, Tiago Luiz Luz Leiria

**Affiliations:** Instituto de Cardiologia - Fundação Universitária de Cardiologia (IC/FUC), Porto Alegre, RS - Brazil

**Keywords:** Anticoagulants / therapeutic use, Atrial Fibrillation, Stroke, Hemorrhage, Medication Adherence, Telemedicine

## Abstract

**Background:**

Atrial fibrillation is responsible for one in four strokes, which may be prevented by oral anticoagulation, an underused therapy around the world. Considering the challenges imposed by this sort of treatment, mobile health support for shared decision-making may improve patients’ knowledge and optimize the decisional process.

**Objective:**

To develop and evaluate a mobile application to support shared decision about thromboembolic prophylaxis in atrial fibrillation.

**Methods:**

We developed an application to be used during the clinical visit, including a video about atrial fibrillation, risk calculators, explanatory graphics and information on the drugs available for treatment. In the pilot phase, 30 patients interacted with the application, which was evaluated qualitatively and by a disease knowledge questionnaire and a decisional conflict scale.

**Results:**

The number of correct answers in the questionnaire about the disease was significantly higher after the interaction with the application (from 4.7 ± 1.8 to 7.2 ± 1.0, p < 0.001). The decisional conflict scale, administered after selecting the therapy with the app support, resulted in an average of 11 ± 16/100 points, indicating a low decisional conflict.

**Conclusions:**

The use of a mobile application during medical visits on anticoagulation in atrial fibrillation improves disease knowledge, enabling a shared decision with low decisional conflict. Further studies are needed to confirm if this finding can be translated into clinical benefit.

## Introduction

Atrial fibrillation (AF) affects 33.5 million people in the world^1^ and is the cause of 28% of strokes.^2^ Prophylaxis with oral anticoagulants (OACs) can reduce the risk of stroke by 60-70%,^3-6^ with a variable risk of bleeding.

AF guidelines recommend the use of the CHA_2_DS_2_-VASc (for stroke) and HAS-BLED (for bleeding) risk scores to recognize those patients who will benefit the most from anticoagulants.^7-10^ More recently, the SAMe-TT_2_R_2_ score^11^ was validated to predict a poor anticoagulation control with coumarins, contributing to the selection of the anticoagulant type. Although many scores are available,^12^ their use should be done with caution. The current European guideline,^8^ for example, recommends the use of bleeding scores to identify modifiable risk factors for major bleeding rather than to contraindicate anticoagulation. Besides, these scores do not take into account patients’ worries, objectives and values, and do not evaluate costs, posology, and frequency of visits to physician and exams, which influence adherence to treatment.^13^ The complexity of such decision process is reflected in the suboptimal number of patients who receive an OAC prescription, maintain target coagulation and adhere to drug treatment.^13-15^

New approaches for the management of chronic diseases have been patient-centered, in which the patient practices shared treatment decision making, leading to improved outcomes and efficacy of the health system.^16,17^ Patients with AF are likely to benefit from these strategies, due to the importance of patient ownership of decisions that require patient action, such as taking the medication and monitoring of treatment.^18^

Mobile health technology, or just mobile health (mHealth) - seems promising in expanding healthcare coverage, facilitating the decision-making process and improving the management of chronic diseases.^17-20^ In 2015, more than 3 billion health app downloads were made worldwide.^21^ It is important that this new technology includes other specific groups, such as the elderly and low-income adults with limited access to mobile communication.^18.22^ In this article, we describe the development of a mHealth application to be used during medical visits, aiming to facilitate the shared decision-making on thromboembolic prophylaxis in AF. The app was tested in low-income patients with low educational attainment by the measurement of disease knowledge before and after its use.

## Methods

### Development of the application

The development staff was composed by a cardiologist, an electrophysiologist, a software developer and a designer.

First, the following fundamental aspects were defined: condition/problem to be approached (thromboembolic prophylaxis in AF); target users/population (patients with AF and low socioeconomic and cultural status); initial application targets (increased knowledge about disease and treatment); situation in which the app would be used (during medical visits); device in which the app would be installed (doctor’s tablet computer) and programming languages (Android and iOS).

A comprehensive literature review was performed, including the main randomized clinical trials, systematic reviews, meta-analyses, and guidelines on AF and OAC, which provided the main scores to be used and relevant information to be conveyed to the users.

**Figure 1:**
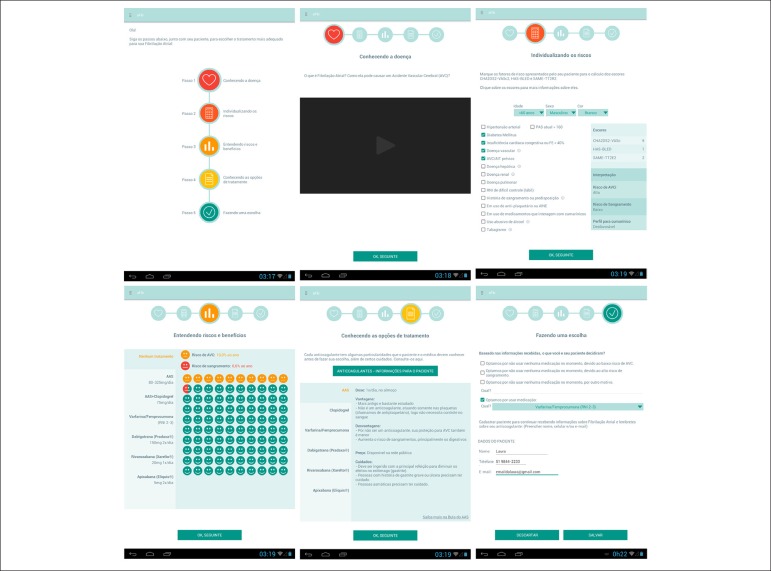
Main screens of the aFib app, developed to help in the shared decision about thromboembolic prophylaxis in atrial fibrillation.

Aiming to translate this information into knowledge to the patient, a simplified navigation through five screens (Figure 1): (1) Knowing the disease - a video about how AF occurs and how it can cause a thromboembolic event; (2) Individualizing the risks - a calculator integrated with the CHA_2_DS_2_-VASc, HAS-BLED and SAMe-TT2R2^11^ scores; (3) Understanding risks and benefits - a screen with pictograms to visualize the percentage of the risk of stroke and bleeding in each treatment option; (4) Knowing the treatment option - a summary of the main characteristics of the drugs available; and (5) Making a choice - the final screen, in which information is saved and the number of patient’s cell phone may be registered to receive information via Short Message Service (SMS).

This navigation format emphasized the main points, providing additional access to more detailed information through the links, according to the users’ needs. For example, in the area of medications, data of posology, approximate costs, advantages and disadvantages of each drug were informed. Also, the official package insert of the drug provided by the Brazilian National Health Surveillance Agency was accessible through a link. Push technology by SMS is a strategy used to enhance the provision of information without overloading the patient with information in only one meeting. In this technology, the patient periodically receives alerts on the importance of adhering to drug therapy and doing some tests, as well as disease information, which can be saved in message box for further reading by the patient.

We opted for a clean and clear design, with a color code for the risks and the use of graphical information whenever possible to complement the written information. Terminology was adapted to the target users. Personal health information were protected by unique identification and secured by cryptography. A privacy policy was presented prior to the use.

### Study design

Intervention study in patients diagnosed with AF in the anticoagulation outpatient service at Porto Alegre Institute of Cardiology, in April and May 2016.

### Population and sample characteristics

The study population comprised patients attending the anticoagulation outpatient service, and the study was carried out during patients’ waiting time for the prothrombin time (PT) test. Before starting their treatment at the anticoagulation outpatient service, each patient receives instructions about AF, the use of OAC, as well as appropriate dose adjustment every 1-3 months. In ten random mornings, all AF patients attending the outpatient center for the PT test were invited for the study and all of them agreed to participate. There was no patient with severe visual disorder, hearing loss or cognitive disorder that would impair patient’s interaction with the app. Patients with one of these conditions would be excluded from the study.

### Pilot study and sample calculation

In the pilot phase, the beta version of the app was used with 10 patients, who gave their feedback to questions about usability, written and visual language, understanding of information, design and adequacy of time for scrolling the screens. Before the appointment, a questionnaire developed by the investigators was administered to measure the mean level of knowledge about AF in this population. This questionnaire sought to evaluate the minimum essential information required for the patient to understand their condition and adhere to the treatment. Patients were asked to answer each of eight statements with “true”, “false” or “don’t know”. All statements were true. Mean number of correct answers was 5.9 ± 1.37 (73% of correct answers). In a previous study conducted at the same service, 64% of patients had adequate knowledge about the therapy.^19^ Considering that the number of correct answers was estimated to increase to 8 (100% of correct answers) after the explanatory intervention, 18 patients were required for a 5% alpha error and a beta error of 90%.

### Outcome measures

After adjustments made after the feedback of the pilot study patients, the app was tested in a sample of 20 patients.

As the primary outcome, we analyzed the scores obtained by the patients in the AF knowledge questionnaire before and after the interaction with the app.

As secondary outcome, we evaluated patients’ scores in the Decisional Conflict Scale in Health (DCSH) by O’Connor,^20^ used to evaluate strategies for shared decision-making in health care.^20,21^ DCSH was validated in Portuguese in 2013 by Martinho et al.^22^ and included questions on uncertainties, knowledge, values and provided support. The total score varied from 0 (no decisional conflict) to 100 (extremely high decisional conflict). Also as a secondary outcome, we analyzed the perceived risk of stroke and bleeding with the use of OAC. Patients were asked if they believed they had a low, moderate, or high risk of each event. This question was repeated after the interaction with the app, and results were compared with the “real” risk, calculated by the CHA_2_DS_2_-VASc and HAS-BLED scores.

### Data analysis

Data were analyzed using the SPSS software version 20.0. Tables of absolute and relative frequencies were used for sample characterization. Normality of data was tested by the Shapiro-Wilk test.

Continuous variables with normal distribution were expressed as mean and standard deviation, and those with non-normal distribution as median and interquartile ranges. Mean knowledge scores about the disease, before and after the intervention, were compared using the paired Student’s t-test and risk perception was compared with the Wilcoxon test. The level of significance was set at p < 0.05.

### Ethical considerations

The study was approved by the Research Ethics Committee of the Institute of Cardiology University Foundation. Privacy, anonymity and confidentiality of data collected were guaranteed, and informed consent form was presented to the patients.

## Results

Mean age of the 20 patients studied was 67.7 years; most patients were men (60.0%), white (83.3%) and lived with their relatives (53.3%). Self-reported educational level was some secondary education in 73.3% of patients, and 33.3% studied less than 4 years. Family income was lower than 2 minimum wages in 53.3% of patients. Most patients (66.7%) used anticoagulants for at least one year. Table 1 summarizes the socioeconomic characteristics and clotting time of the study population.

**Table 1:** Socioeconomic characteristics of the population and time in anticoagulation therapy

Characteristics	
Age (years)	67.7 ± 9.4
Male sex (%)	60
White (%)	83.3
**Who patients live with**	
Alone (%)	16.7
Companion (%)	26.7
Family (%)	53.3
Institutionalized (%)	3.3
**Schooling years**	
0-4 years (%)	33.3
5-8 years (%)	40
> 8 years (%)	26.7
**Family income**	
4-10 minimum wages (%)	26.7
2-4 minimum wages (%)	20
< 2 minimum wages (%)	53.3
**Time in anticoagulation therapy**	
< 1 month (%)	13.3
1 - 11 months (%)	13.3
1-5 years (%)	33.3
> 5 years (%)	33.4
Not in current use	3.3

Table 2 shows the prevalence of the main risk factors included in the CHA2DS2-VASc, HAS-BLED and SAMe-TT2R2 scores, and the mean ratings obtained by the patients in these scores. The most prevalent comorbidities were arterial hypertension (80%), diabetes *mellitus* (30%), and heart failure (30%). With respect to other factors that may influence the risk of bleeding and anticoagulation, the most common factors were the use of medications that interact with coumarins (43.3%), and the use of antiplatelet or anti-inflammatory drugs (26.7%). Most patients (86.6%) had a CHA2DS2-VASc score equal to or greater than 2 and 76.6% of patients had a SAMe-TT2R2 score equal to or greater than 2.

**Table 2:** Prevalence of the variables present in the CHA_2_DS_2_-VASc, HAS-BLED and SAMe-TT2R2 scores and average scores

Systemic arterial hypertension (%)	80
Systolic blood pressure > 160 mmHg (%)	10
Diabetes Mellitus (%)	30
Congestive heart failure and ejection fraction < 40% (%)	30
Cardiovascular disease (%)	23.3
Stroke or transient ischemic accident (%)	16.7
Liver disease* (%)	0
Kidney disease † (%)	6.7
Pulmonary disease (%)	16.7
Labile or difficult-to-control INR ‡ (%)	23.3
History of or predisposition to major bleeding (%)	16.7
Use of antiplatelet or anti-inflammatory agents (%)	26.7
Use of medications that interact with coumarins (%)	43.3
Abusive use of alcohol (%)	3.3
Smoking (%)	10
CHA_2_DS_2_-VASc ≥ 2 § (%)	86.6
CHA_2_DS_2_-VASc per score (%)	
0	3.3
1	10
2	23.4
3	23.4
4	20
5	13.3
7	3.3
8	3.3
Mean CHA_2_DS_2_-VASc	3 ± 1.8
Mean HAS-BLED	2 ± 1.2
SAMe-TT2R2 ≥ 2 // (%)	76.6

* Chronic liver disease (e.g.: cirrhosis), or biochemical evidence of significant liver dysfunction (bilirubin > 2 - 3 times the upper level, transaminase or alkaline phosphatase > 3 times the upper level);

† Chronic hemodialysis, kidney transplant, serum creatinine > 2.2 mg/dl;

‡ in the target range < 60% of times;

§ A score ≥ 2 indicates the necessity of anticoagulation;// A score ≥ indicates patients who require additional interventions to achieve an acceptable anticoagulation control with coumarins.

**Figure 2:**
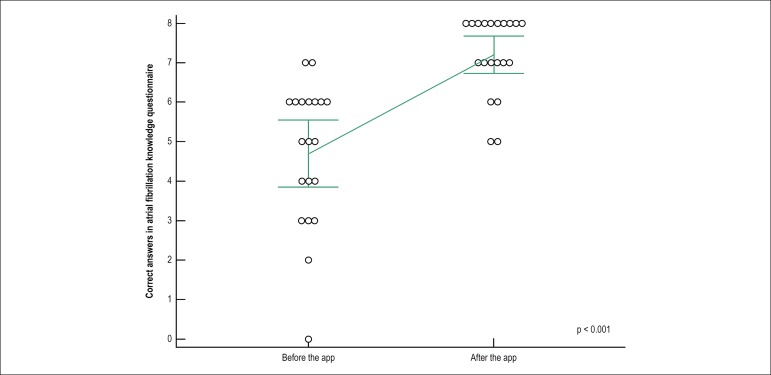
Mean number of correct answers in the questionnaire about the disease before (4.7) and after (7.2) the intervention, compared by the paired-sample t test, indicating a significant increase in patients’ knowledge after interacting with the application. Error bars indicate standard deviations, and circles represent the score of each patient.

The number of correct answers in the disease knowledge questionnaire significantly increased after the interaction with the application, from 4.7 (± 1.8) to 7.2 (± 1.0), p < 0.001. Figure 2 depicts the mean number of correct answers before and after the interaction.

DCSH administered to the patients after selecting the therapy with the aid of the app resulted in an average of 11 ± 16/100 points.

**Figure 3:**
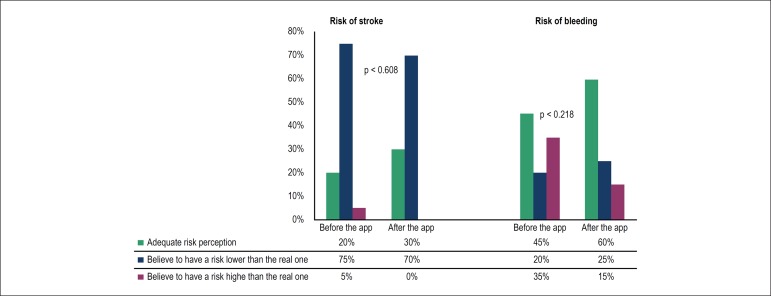
Risk perception of stroke and bleeding by the patients before and after interacting with the application compared with the real risk, calculated by the CHA_2_DS_2_-VASc and HAS-BLED scores, showing a non-significant increase in the adequate perception of the risk. Comparisons were performed by the Wilcoxon test.

Regarding risk perception, before interacting with the app, 20% of patients had an appropriate perception of their risk of stroke, and 75% believed to have a risk lower than the real risk. After the interaction, adequate perception increased to 30%, with a non-significant p-value (0.608). With respect to the risk of bleeding, before using the app, 45% of patients had a correct perception and 35% believed they had a higher risk than the real one. After using the app, there was a non-significant increase (0.218) in the adequate perception for 60% of patients. Figure 3 depicts variations in risk perception.

## Discussion

The development of mHealth apps for specific populations and health problems is viable and should be stimulated. This study with low income and low educational level patients demonstrated increased knowledge about AF and anticoagulation after the use of the app, enabling a shared decision-making about anticoagulation, with low decisional conflict. However, the perception of stroke and bleeding risk was not affected by the application use.

Thromboembolic prophylaxis in AF is a global problem. It is generally underused, of difficult management and known to be prone to poor adherence.^23^ One of the proposed strategies to optimize the use of OAC is the shared decision-making, which is currently recommended in the guidelines as part of an integrated management of the disease, and a clinical performance indicator.^8,24^ Patients’ understanding of the therapy and their individual risk-benefit analysis is crucial in this process.^25^ Nevertheless, there are significant gaps in this knowledge, even in patients treated for years.^18^

Several studies have mentioned instruments that facilitate shared decision-making strategies of anticoagulation in AF, by means of behavior change and patients’ education using leaflets, and interventions using videos and softwares. A Cochrane meta-analysis published in 2013 reviewed these studies, and concluded that there is no sufficient evidence that evaluate the impact of these strategies on the International Normalized Ratio in therapeutic range (TTR, time in therapeutic range).^26^ Another recent review concluded that decision-making strategies with patients’ participation are powerful tools to improve the management of AF and that these instruments should be developed and tested.^18^ Subsequently, the TREAT study, a randomized, controlled study of behavioral intervention in patients who had recently initiated warfarin, showed a significant improvement of TTR in six months, compared with usual care.^27^ Another study involving a multidisciplinary intervention for patients with AF, which included a decision support software, and was conducted and supervised by nurses and cardiologists, respectively, demonstrated a significant reduction in the number of cardiovascular deaths and hospitalization (14.3 vs. 20.8%; risk ration of 0.65; 95% CI 0.45-0.93).^28^

These interventions are based on the premise that the healthcare professional is responsible for the provision of essential information to the patient and for stimulating the patient to search for knowledge. In this context, technology shows up as an allied, by improving information access, organization, transmission and retention. In particular, mobile technology introduces a new era of health care, by bringing care closer to the patient and allowing a better doctor-patient interaction.

In this rapidly expanding market, in 2015, there were 45,000 mHealth publishers and more than 3 billion mHealth app downloads.^29^ Current evaluations are, in general, favorable. A recent analysis of the American Heart Association on mHealth and cardiovascular disease prevention included 69 apps for weight loss, increase in physical activity, smoking cessation, glycemic control, hypertension and dyslipidemia. Despite heterogeneous, positive results were found for the proposed behavioral changes, and future studies using more rigorous methodology, more diversified samples and a long-term follow-up were suggested to evaluate the duration of the effects.^30^

With respect to the target populations, the literature highlights the necessity of these technologies to encompass other specific populations - older subjects with age-related changes (e.g. reduced vision or mobility), minorities in need of culturally sensitive contents and interventions, and low-income adults with inconsistent access to mobile communication.^30-32^

AF is a largely explored subject in mHealth. Most studies have reported the use of home monitoring devices for heart rate. With regards to patients’ education, the American Heart Association and the European Society of Cardiology have high-quality applications and web materials in English that help in the shared decision-making process.^33,34^ There are also many risk calculation methods available for the clinical practice. However, neither the development process nor the evaluation of these apps is described in the literature. Also, we have not found any support instrument for shared decision-making in AF, be it in mHealth or in other media.

A strength of our study was the development of the app based on evidence, taking into account many factors mentioned in guidelines of shared decision making and care of anticoagulated patients.^25,35^ The level of patients’ previous knowledge was analyzed, and the learning style was adequate to their preferences of terminology and navigability. The amount and detail of information was adjusted, and could be increased or reduced, according to each individual’s understanding.

Another advantage was the fact that patients’ evaluation could be saved for further analyses by other professionals, indicating the role of the instrument as a bridge in the multidisciplinary care. In an integrated outpatient service, for example, the patient could watch the video and have their risk factor evaluated during the screening process and focus on treatment during the medical visit.

In addition, the selected population was appropriate for implementing a shared decision strategy. Most patients had a SAMe-TT2R2 score equal to or greater than two, suggesting a lower probability to maintain anticoagulation at acceptable levels with coumarins and hence a greater necessity for strategies for an adequate control.

Results of the analysis of patients’ risk perception showed how this understanding is inappropriate and requires attention. Most patients believed they had a stroke risk lower than the calculated and one third of patients believed they had a bleeding risk with the use of OAC higher than the calculated. Other studies showed similar results on awareness of the risk of stroke.^36,37^ Such inadequate understanding may lead to poor treatment adherence, since patients do not perceive themselves to be at risk for thromboembolic events and also believe they have a high risk of bleeding using the medication. After interacting with the app, no significant change in risk perception was observed. In attempt to improve such perception, the following observation was added to the second version of the app, currently under test: “This risk is considered LOW/INTERMEDIATE/HIGH”, with a color code to each level of risk (green/yellow/red), together with the percentages exhibited on the screen “Understanding risks and benefits”.

Several limitations are inherent to the development of an instrument that utilizes a relatively new technology for our population. Although the screen size, the visual communication methods and the terminology had been carefully considered, they still can be inadequate for some patients. Besides, even though the information provided to the patients had been adapted to the patients, the fact that it had been excessive in some cases and not maintained after some months cannot be ruled out. It is expected that the continuous provision of information by SMS compensate part of this issue. Besides, the interaction with the app may be repeated in other visits whenever necessary.

The small number of patients studied may also be questioned. Nevertheless, in studies evaluating the usability of apps, the number of subjects involved is usually small and shown sufficient.^38^ Another current limitation is the necessity of a long-term evaluation of the outcomes, such as the TTR, adherence and occurrence of thromboembolic events and bleeding. This limitation is expected to be eliminated with a randomized intervention study, by using the app in the care of our patients attending the anticoagulation outpatient service and comparing the results with the care currently provided.

## Conclusions

The use of the mHealth app during the medical visit about anticoagulation in AF improves disease knowledge and the treatment of low-income patients with low educational level, enabling a shared decision with low decisional conflict. Further studies are needed to confirm whether such improvement can be translated into hard outcomes.
